# The Molecular Mechanism and Effects of Root Pruning Treatment on Blueberry Tree Growth

**DOI:** 10.3390/plants14152269

**Published:** 2025-07-23

**Authors:** Liwei Chu, Chengjing Shi, Xin Wang, Benyin Li, Siyu Zuo, Qixuan Li, Jiarui Han, Hexin Wang, Xin Lou

**Affiliations:** 1 School of Life and Health, Dalian University, No. 10 Xuefu Street, Dalian 116622, China; chuliwei@dlu.edu.cn (L.C.);; 2 Institute of Modern Agricultural Research, Dalian University, No. 10 Xuefu Street, Dalian 116622, China

**Keywords:** root pruning, root growth, transcriptome, vascular tissue development

## Abstract

Root pruning can promote the transplanting of young green plants, but the overall impact of pruning on root growth, morphology, and physiological functions remains unclear. This study integrated transcriptomics and physiological analyses to elucidate the effects of root pruning on blueberry growth. Appropriate pruning (CT4) significantly promoted plant growth, with above-ground biomass and leaf biomass significantly increasing compared to the control group within 42 days. Photosynthesis temporarily decreased at 7 days but recovered at 21 and 42 days. Transcriptomics analysis showed that the cellulose metabolism pathway was rapidly activated and influenced multiple key genes in the starch metabolism pathway. Importantly, transcription factors associated with vascular development were also significantly increased at 7, 21, and 42 days after root pruning, indicating their role in regulating vascular differentiation. Enhanced aboveground growth was positively correlated with the expression of photosynthesis-related genes, and the transport of photosynthetic products via vascular tissues provided a carbon source for root development. Thus, root development is closely related to leaf photosynthesis, and changes in gene expression associated with vascular tissue development directly influence root development, ultimately ensuring coordinated growth between aboveground and belowground parts. These findings provide a theoretical basis for optimizing root pruning strategies to enhance blueberry growth and yield.

## 1. Introduction

The quality of seedling growth directly influences subsequent growth and yield, rendering it an essential factor in agricultural production [1]. Nutrient bowl seedling cultivation is widely recognized as an effective method for seedling cultivation [2]. Although nutrient pot nurseries can maintain the relative integrity of the root system, they also present deficiencies such as reduced capillary roots as well as coiled, aged, and deformed root systems. Therefore, bare-root seedling transplantation remains widely practiced [3], with root pruning being a common technique to facilitate seedling transplantation and transportation [4]. Root pruning is a common artificial root disturbance technique used in seedling nurseries, whereby the regeneration and optimization of the plant root system are stimulated by subtracting a portion of the seedling’s primary root, thereby improving plant growth performance and health [5]. Previous studies on grafted thin-shelled walnut seedlings have shown that an optimal root-cutting ratio could enhance root length, diameter, and total dry weight, thereby improving the quality indices of seedlings [6]. This research also explored the effects of various root pruning methods by examining characteristics including the root surface area, root length, root volume, number of roots, and root diameter. The results demonstrated that appropriately proportioned root pruning could improve the root morphology of the plant, which in turn enhances the quality indicators of the seedlings [7]. It has been reported that properly proportioned root pruning improved water use efficiency in corn plants [8]. Root pruning is an effective method for improving plant photosynthetic traits, increasing fruit yield [9,10,11], and promoting plant growth [12].

Root pruning, as an agricultural management practice, exerts a significant effect on plant growth and development, a process that involves complex physiological phenomena and morphological changes [13,14]. First, root pruning stimulates a range of physiological responses in plants, most notably changes in the phytohormone content. Interestingly, it has been reported that root pruning could induce significant changes in the contents of phytohormones, such as increases in the contents of indole-3-acetic acid (IAA), jasmonic acid (JA), and abscisic acid (ABA) in roots, which are usually involved in plant growth and defense responses [15]. Moreover, under stress or disturbance conditions, such as root pruning, the plant antioxidative enzyme system is activated to scavenge the excessive reactive oxygen species (ROS) [16]. Overall, changes in the contents and activities of phytohormones and enzymes contribute to the recovery of the pruned roots or the initiation of lateral roots following pruning [17,18].

In addition, root pruning affects the lignin and cellulose content of the root system. Lignin, an important secondary metabolite, enhances the mechanical strength of the cell wall and improves the stability of the root system [19]. After pruning, the root system increases lignin synthesis in response to wounds and possible pathogen invasion, thus forming a protective barrier around the wound [20]. At the same time, cellulose synthesis may be regulated to maintain cell wall structure and function. Morphologically, the significant enlargement of vascular tissue that root pruning induces is the result of the high expression of vascular-related genes around the edge of the root incision [21]. Vascular tissue is the main structure responsible for material transport in plants, including xylem and phloem. After pruning, vascular tissues undergo a series of adaptations to accelerate wound healing and root recovery. In this respect, it has been shown that xylem conduits may become more developed to improve the efficiency of water and inorganic salt transport [22,23,24], while phloem sieve tubes may enhance their ability to transport the products of photosynthesis to provide an adequate energy and material base for root growth [25,26]. Furthermore, root pruning exerts substantial effects on the development of vascular tissues. During the post-pruning recovery process, vascular tissues undergo a series of complex processes such as cell division, differentiation, and programmed death to form new ducts and sieve tube systems. These newly formed vascular tissues not only demonstrate refined transport efficiency but also exhibit enhanced adaptability to environmental changes, contributing to improved plant resistance and productivity. Although root pruning techniques are widely applied in crop cultivation management, current research still faces two major limitations: first, the physiological response mechanisms triggered by pruning (such as dynamic changes in photosynthesis and adjustments in carbon-nitrogen allocation) and their association with molecular-level events (such as vascular tissue regeneration) remain fragmented and lack systematic integration. Second, the specific differences between woody and herbaceous plants in root regeneration remain unclear, particularly whether shallow-rooted woody plants (such as blueberries) possess unique molecular pathways and structural reconstruction patterns for regeneration, which remains undecided. These two limitations constrain the precise application of root pruning techniques in perennial woody economic crops.

Seedling nurseries are important for the development of the blueberry industry. As a woody plant characterized by fibrous, shallow, and relatively weak root system, blueberry plants have high requirements for growing soil media, and the substrate potting method has gradually become a prevalent cultivation trend [27]. However, with the growth of potted blueberries, the root system growth is constrained by the container, leading to significant entanglement against the pot walls and a resultant insufficient supply of plant nutrients. Therefore, practical management necessitates providing the root system with more space to grow (e.g., change to larger-size containers) or employing appropriate techniques to remove redundant roots (e.g., root pruning) to promote root regeneration, which in turn affects plant growth.

Root pruning is an important management measure in blueberry nurseries; therefore, analyzing the molecular mechanisms of blueberry root pruning can improve blueberry cultivation efficiency. The present study aims to investigate the regulatory mechanism of root pruning regarding blueberry root development at the molecular level, which holds great scientific significance for revealing the molecular basis by which partial primary root removal promotes the development of lateral roots and provides important scientific data for the propagation of woody plants.

The selection of Vaccinium corymbosum as a model system is based on its unique biological constraints, economic importance, and unresolved molecular mechanisms. First, unlike deep-rooted woody perennials, blueberries have shallow, fibrous root systems with extremely low root hair development. This morphological specialization makes them highly susceptible to root-restricted stress in container cultivation, thereby enhancing phenotypic responses to pruning interventions [28]. Second, as the world’s most valuable berry crop [29], China’s rapidly developing blueberry industry faces a 30% yield gap due to transplant losses, a gap that could be addressed through optimized root management. Finally, while root pruning can enhance yields in Rosaceae crops, little research has been conducted on transcription regulatory factors associated with vascular bundle regeneration in Ericaceae plants, leaving a critical knowledge gap for breeding programs aimed at developing stress-tolerant root systems.

## 2. Materials and Methods

### 2.1. Plant Material and Experimental Design

The experimental materials were selected from 6-month-old cuttings of the blueberry variety ‘Eureka’ provided by Dalian Senmao Modern Agriculture Co., Ltd. in Dalian, China. The trial was conducted in the greenhouse of Dalian University Cooperative Experimental Base from April to June 2023. In April 2023, the average daily temperature ranged from 8 °C to 16 °C, with an average relative humidity of 68%. Under clear weather conditions, the photosynthetically active radiation (PAR) at noon can reach 12,000 μmol/(m^2^·s). The experimental photoperiod was 14 h/10 h (light duration/dark period). A one-way randomized block design was applied, setting no root pruning as the control group and six treatment groups involving the pruning of 20%, 40%, 50%, 60%, 80%, and 100% of the root system, with a total of seven experimental groups and five experimental seedlings in each group. In April 2023, 35 blueberry seedlings cultivated in the self-developed root culture observation device, exhibiting good growth conditions and having been cultivated in a custom-designed root culture observation device (where root growth had reached the glass plate edges, indicating restriction), were selected for root pruning treatments (pruning only once) centered on the root node, at both sides and the bottom of the root clipping treatments to maintain the root width of the root in equal proportions to reduce the root system by 20% (CT2), 40% (CT4), 50% (CT5), 60% (CT6), and 80% (CT8). The 100% pruning treatment involved the retention of only the primary root 1–2 cm below the root node. Calculate the area to be cut according to the schematic diagram in Figure S3 of Supplementary Information 2. Use an ethanol-disinfected blade to cut at a 90° angle at the root cutting site.

The custom-designed root culture observation device consisted of a 30 cm × 30 cm glass plate clamped on both sides, separated by a 1 cm glass sealing strip in the middle, and three 5 cm dovetail clamps were used on both sides to clamp the upper, middle, and lower parts, respectively, with aluminum-plated reflective film on the outer cover to block out the light. The schematic diagram is shown in Figure S2 in Supplementary Information 2. This study utilized a growing medium composed of perlite, peat moss, and coconut coir mixed at a 1:1:1 volume ratio, which was subjected to high-temperature sterilization treatment (121 °C for 30 min) to eliminate pathogens and weed seeds. Its physical and chemical properties are as follows: total porosity of 85–90%, water-holding capacity of 60–75%, combining good aeration and water retention, pH of 5.5–6.5, slightly acidic, organic matter content of 30–40%, and electrical conductivity of 0.3–0.5 mS/cm (unfertilized). This formulation aligns with standard blueberry cultivation practices, providing optimal aeration, acidity, and water retention for fibrous root development. The nutrient solution was watered twice a day during the experimental period, based on the Hoagland and Arnon nutrient solution [30], and supplemented with trace elements from other common formulas within a pH range of 4.0–4.5.

### 2.2. Individual Morphometry

At 7, 21, and 42 days after the experimental treatments, the glass panel on one side of the root growth observation device was opened, and a scanner (SinocrystalScanMaker 9800XL, Shanghai, China) was used to scan and record the root growth status of the seedlings in each group of experimental seedlings, with three blueberry plants selected from each group. The height and basal stem of blueberry plants were measured using a steel tape measure (accuracy 0.1 cm) and vernier caliper (accuracy 0.01 mm) at 7 d, 21 d, and 42 d after root pruning treatment, respectively. The number of leaves on each plant were recorded, selecting three biological replicates for each group. Leaves were scanned with a scanner at the end of the treatment and, subsequently, the average leaf area was calculated using Adobe Photoshop 2019. The calculation formula was as follows [31]:

$$Leaf\;area({cm}^{2})=Number\;of\;leaves\times Average\;leaf\;area$$

Forty-two days after the end of the treatment, three blueberry plants exhibiting uniform growth were randomly selected from each treatment group. The leaves and stems were rinsed, dried, and weighed at 0.5 g each using a balance with a precision of 0.001 g. The root system of each blueberry plant was washed with distilled water, wiped dry, and weighed using a balance with a precision of 0.0001 g to obtain 0.2 g, with three replicates. Subsequently, the weighed blueberry leaves, stems, and root systems were labeled and put into the oven for inactivation at 110 °C for half an hour and then baked at 75 °C until they reached a constant weight. The dry weight was then recorded separately. The calculation formula was as follows [32]:

Water Content(%)=(Fresh weight−Dry weight)/Dry weight×100

### 2.3. Determination of Photosynthetic and Chlorophyll Fluorescence Parameters

Photosynthetic parameters including the photosynthetic rate (Pn), transpiration rate (Tr), stomatal conductance (Gs), and substomatal cavity CO_2_ concentration (Ci) of the same functional leaves of blueberry plants in the root pruning group and the control group were measured using the Li-6400 XT Portable Photosynthesis (Beijing ecotek Company, Beijing, China) Instrument between 9:00 a.m. and 11:00 a.m. at 7, 21, and 42 d after the experimental treatments. Photosynthesis parameters were measured on three biologically independent plants in each treatment group. For each plant, three mature leaves (located at the third to fifth nodes from the top) were selected as technical replicates, and measurements were taken between 9:00 and 11:00 a.m. on a clear day.

The chlorophyll fluorescence parameters, including maximal photochemical efficiency (F_v_/F_m_), photosystem II (PSII), and nonphotochemical quenching (NPQ), were assessed using the Li-6400 XT Portable Photosynthesis Instrument at the same time as the photosynthetic measurements.

### 2.4. RNA Extraction, cDNA Library Construction, and Transcriptome Sequencing

RNA was extracted from roots and leaves at different developmental stages from the pruning group and the control group. All freeze-dried samples were ground into powder in liquid nitrogen, and 50 mg of powder was used for total RNA extraction using an RNA extraction kit (Beijing Adelaide Biotechnology Co., Ltd., Beijing, China). The RNA concentration, RIN value, 28S/18S ratio, and fragment size were measured using an Agilent 2100 Bioanalyzer (Beijing, China) to assess RNA integrity. RNA purity and concentration were determined using a NanoDrop™ UV-visible spectrophotometer (Thermo Fisher Scientific Inc., Shanghai, China) based on the OD260/280 ratio.

The DNBSEQ platform was used for library construction and quality control. After the samples passed the test, the library was constructed according to the following steps: enrich mRNA with magnetic beads with Oligo (dT) and add the fragmentation buffer to interrupt mRNA. The first cDNA strand was synthesized with six-base random hexamers using mRNA as a template, and then the second cDNA strand was synthesized by adding buffer, deoxynucleotide mix (dNTPs), Ribonuclease H (RNaseH), and DNA polymerase I. Then, double-stranded cDNA was purified using a kit (Thermos Fisher Scientific Inc., Shanghai, China). The purified double-stranded cDNA was terminal-repaired, and a tail was added with a sequencing joint connected. Finally, PCR amplification was performed to construct the sequencing library. After the library was constructed, the insert range of the library was examined using an Agilent 2100 Bioanalyzer. The ABI StepOnePlus Real-Time PCR System was used to quantify the library concentrations. After passing the quality inspection, sequencing was performed using an Illumina platform sequencer (Beijing, China).

### 2.5. Functional Analysis of Differentially Expressed Genes

The predicted new genes were functionally annotated using the NR, GO, and KEGG databases to obtain detailed descriptions of the new genes. Cleaned read sequences were aligned to the reference genome *Vaccinium darrowii* v1.2 using HISAT2 v2.2.1. Transcriptome reconstruction was performed for each sample using StringTie. Subsequently, CPC was used to predict the protein-coding potential of novel transcripts, and novel transcripts identified as potential protein-coding transcripts were added to the reference gene sequence to generate a comprehensive reference sequence, which was then used for subsequent analyses. Differential expression analysis was performed using DESeq2, with a threshold of |log2Fold Change| > 1 and *q*-value < 0.001 to identify differentially expressed genes. Finally, GETORF was used to detect the open reading frames (ORFs) of each differentially expressed gene (DEG), and the ORFs were compared with transcription factor protein domains (from PlantTFDB) using hmmsearch. Then, the transcription factor encoding capability characteristics of DEGs were analyzed based on the transcription factor family characteristics described in PlantfDB.

### 2.6. Identification of Transcription Factors Related to Vascular Development

To systematically analyze the regulatory mechanisms underlying root pruning-induced vascular tissue development, this study screened and validated key TFs related to vascular tissue differentiation in blueberry based on public databases and homology comparison strategies. The specific process was as follows: 1. Transcription factor data sources: reported vascular tissue development-related transcription factor families were downloaded as protein sequences from NCBI RefSeq (Visited from 15 January 2025 to 19 March 2025; https://www.ncbi.nlm.nih.gov/) and Phytozome v13 (Visited from 10 January 2025 to 3 March 2025; https://phytozome-next.jgi.doe.gov/) (e.g., NAC, AP2/ERF, MYB, HD-ZIP) for model plants such as *Arabidopsis thaliana* and *Populus trichocarpa*. The predicted transcription factor sequences were extracted by integrating the gene annotation file of the blueberry (*Vaccinium corymbosum*) reference genome *Vaccinium darrowii* clone NJ8810/NJ8807 v1.2 genome sequence. 2. Homology comparison and candidate gene screening involved the local alignment of the blueberry genome against model plant development-related TFs using BLAST+ v2.13.0 as query sequences, based on the following thresholds: E-value ≤ 1 × 10^−5^, coverage ≥ 60%, and sequence identity ≥ 40%. A Neighbor-Joining phylogenetic tree was constructed using MEGA 11. The known functional TFs of *Arabidopsis thaliana* and *Populus trichocarpa* were used as references to cluster the blueberry candidate genes and infer their potential functions. Screening for orthologs focused on TFs that were highly expressed in vascular tissues (e.g., Arabidopsis *VND7*, *SND1*).

### 2.7. Quantitative RT-PCR Analysis

Gene expression in developing blueberry leaves and root systems was verified using qRT-PCR. Total RNA isolated from developing blueberry leaves and roots at 7, 21, and 42 days after root pruning was reverse-transcribed, and cDNA was synthesized using the NovoStart^®^ Plus All-in-one 1st Strand cDNA Synthesis SuperMix kit according to the manufacturer’s instructions. The cDNA was diluted to 1 ng μL^−1^ with ddH_2_O for further examination. Gene-specific primers were designed using Oligo7, and the *Actin* endogenous reference gene was used as an endogenous reference using the NovoStart^®^ SYBR qPCR SuperMix Plus kit. The primers for each single gene detected are detailed in Supplementary Table S1. Three technical replicates were performed for each sample and data were analyzed using the 2^−ΔΔCT^ method.

### 2.8. Data Processing and Statistical Analysis

Raw data were analyzed using Excel 2016 (Microsoft Corp, Albuquerque, NM, USA). Statistical analysis was performed using SPSS Statistics version 25.0 (IBM Corporation, Armonk, NY, USA) with analysis of variance (ANOVA), and significant differences were determined using the Least Significant Difference (LSD) post hoc test at a significance level of *p* < 0.05. Heatmaps were created with TBtools-II.

## 3. Results

### 3.1. The Effect of Different Degrees of Root Pruning on Blueberry Plant Growth and Development

**Figure 1:**
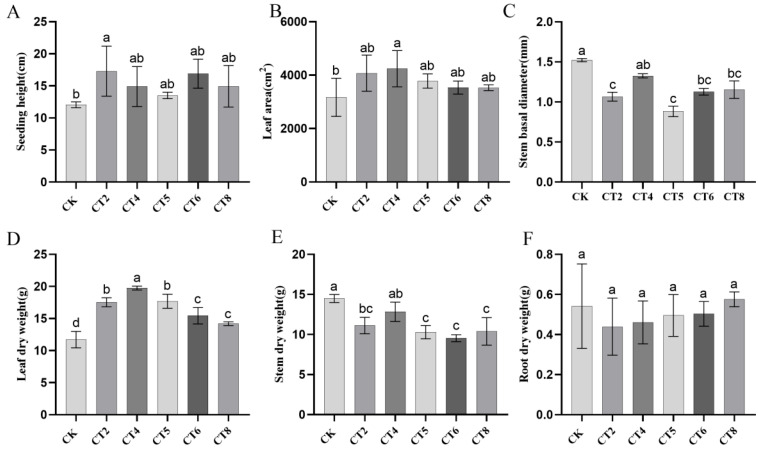
Effects of root pruning on plant physiology in blueberry plants. Plant height (**A**), leaf area (**B**), stem basal diameter (**C**), leaf dry weight (**D**), stem dry weight (**E**), and root dry weight (**F**) 56 days after root pruning. Data are presented as the mean ± standard error (each group includes three biologically independent plants). Different lowercase letters indicate significant differences between groups (one-way ANOVA, *p* < 0.05). CK, control group (no root pruning); CT2, 20% root pruning; CT4, 40% root pruning; CT5, 50% root pruning; CT6, 60% root pruning; CT8, 80% root pruning.

At 56 days after root pruning, the plant height of CT2 was 43.6% higher than that of the control group, and the leaf area of CT4 increased by 33.8% compared to that of the control. Other treatments showed non-significant increases in plant height and leaf area (Figure 1A,B; Table S2). Stem basal diameters were significantly smaller than the control, except for CT4, which was reduced by 30%, 12.8%, 29.6%, 25.9%, and 24.3%, respectively (Figure 1C).

Fifty-six days after root pruning treatment, the leaf dry weight was significantly increased by 49.7%, 68.7%, 51.1%, 31.9%, and 21.3% in CT2, CT4, CT5, CT6, and CT8, respectively, compared to the control. The stem dry weight decreased significantly in all treatment groups except for CT4, with reductions of 23.3%, 11.4%, 29%, 34.2%, and 28.3%, respectively. The total above-ground biomass increased significantly by 24.4% only in CT4 compared to CK, while the other treatment groups did not show any significant difference (Figure 1D,E). The root dry weight was lower than that of the control among treatment groups, although none of these differences were statistically significant (Figure 1F). These results indicated that the CT4 treatment group significantly facilitated the above-ground growth of blueberry plants, mainly manifested as a significant promotion of leaf biomass accumulation.

### 3.2. Growth and Development of Blueberry Plants Under CT4 Root Pruning

**Figure 2:**
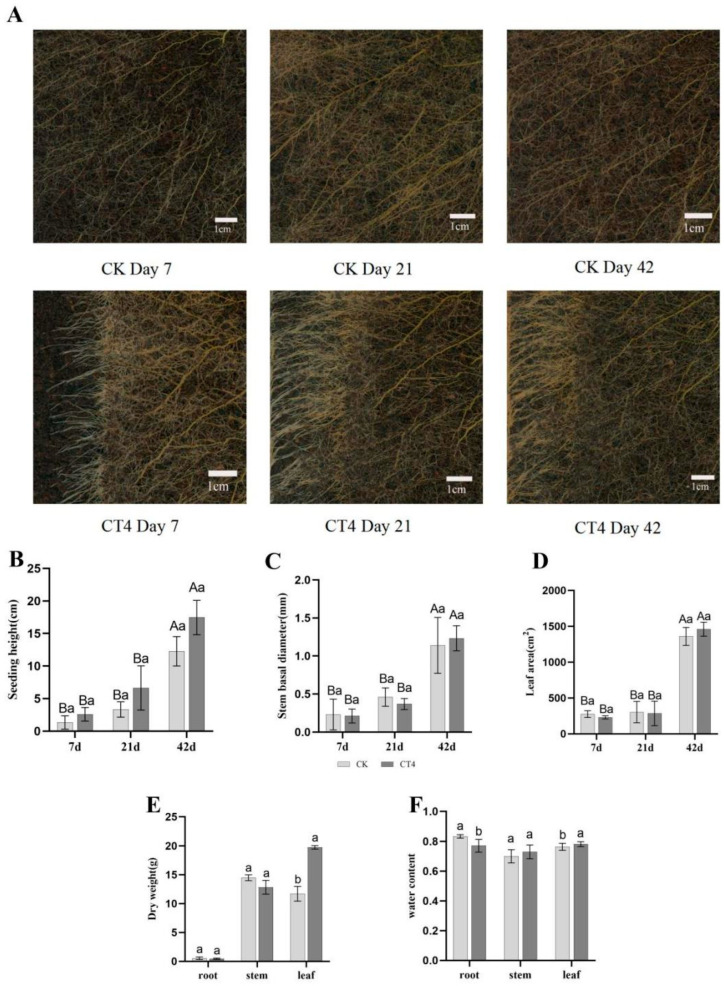
Morphological and biomass changes in the root systems of blueberry plants after root pruning. Phenotypic changes related to the growth and development of CT4 blueberry plants on days 7, 21, and 42 after root pruning (**A**). (CK) were the control, CT4 was 40% root pruning. Seeding height (**B**), stem basal diameter (**C**), and leaf area (**D**) of blueberry plants 7, 21, and 42 days after root pruning. Dry weight (**E**) and water content (**F**) of roots, stems, and leaves, respectively, on day 42 of root pruning. Data are presented as the mean ± standard error (each group contains three biologically independent plants). Different capital letters in the figure indicate significant differences between the same treatment at different time points (one-way analysis of variance, *p* < 0.05). Different lowercase letters indicate significant differences between different treatments at the same time point (*p* < 0.05). Vertical bars indicate standard error.

To further analyze the phenotypic alternations associated with the CT4-promoted growth and development of blueberry plants, various morphological indices were measured at days 7, 21, and 42 post-root pruning (Figure 2A; Figure S1). Following root pruning treatment, the plant height, stem basal diameter, and leaf area increased gradually, but no significant difference was found between CT4 and the control group (Figure 2B–D; Table S3). A total of 42 days after root pruning, the leaf dry weight of CT4 was 68.66% higher than that of the control. However, there was no significant difference in root and stem dry weights between CT4 and the control (Figure 2E). The root water content of CT4 root pruning treatment was significantly lower than that of the control by 7.48%, while the leaf water content of CT4 treatment was significantly higher than that of the control by 2.34%. The stem water content of CT4 root pruning treatment was not significantly different from that of the control (Figure 2F).

### 3.3. Dynamic Changes in the Photosynthesis of Blueberry Plants Under CT4 Root Pruning Degree

**Figure 3:**
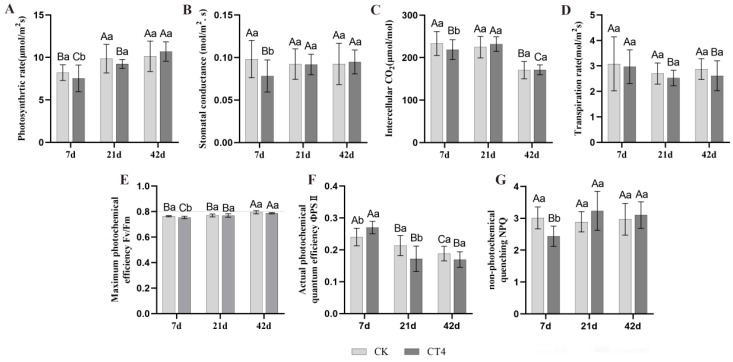
Photosynthetic function of blueberry plants after root pruning. Photosynthetic rate (**A**), stomatal conductance (**B**), intercellular CO_2_ (**C**), transpiration rate (**D**), F_v_/F_m_ (**E**), ΦPSII (**F**), and NPQ (**G**) on days 7, 21, and 42 after root pruning. Data are presented as the mean ± standard error (each group contains three biologically independent plants). Different capital letters in the figure indicate significant differences between the same treatment at different time points (one-way analysis of variance, *p* < 0.05). At each time point, between-group differences were analyzed using unpaired Student’s *t*-tests and corrected using Bonferroni correction. Different lowercase letters indicate significant differences between different treatments at the same time point (*p* < 0.05). Vertical bars indicate the standard error.

The net photosynthetic rate of blueberry plants was significantly increased over the duration of root pruning treatments. The root pruning treatment significantly reduced the net photosynthetic rate only 7 days after pruning. The net photosynthetic rate of blueberry plants reached 8.21 μmol/(m^2^·s) without root pruning and decreased to 7.54 μmol/(m^2^·s) with CT4, representing an 8.16% reduction compared to the control. There was no significant difference in the net photosynthetic rate at 21 and 42 days after root pruning compared to the control (Figure 3A; Table S4).

The extent of stomata opening is reflected by stomatal conductance and directly affects photosynthesis, respiration, and transpiration in the plant. The root pruning treatment significantly reduced stomatal conductance only 7 days after pruning. In the absence of root pruning, the stomatal conductance of blueberry plants reached 0.098 mol/(m^2^·s), and dropped to 0.078 mol/(m^2^·s) under CT4 treatment, representing a 20.41% decrease compared to the control. There were no significant differences in transpiration rates at 21 and 42 days after root pruning compared to the control (Figure 3B).

The intercellular CO_2_ content indicates CO_2_ storage in plant cells, directly affecting the availability of substrates for photosynthesis and the uptake of atmospheric CO_2_ through guard cells. The root pruning treatment significantly reduced the intercellular CO_2_ concentration only 7 days after pruning. In the absence of root pruning, the intercellular carbon dioxide concentration in blueberry plants reached 233.30 μmol/mol and dropped to 219.01 μmol/mol with CT4 treatment, representing a 6.13% decrease compared to the control. There was no significant difference in the intercellular CO_2_ concentration 21 and 42 days after root pruning compared to the control (Figure 3C).

Water usage and transport in plants can be determined via the transpiration rate. After root pruning for 7, 21, and 42 days, there was no significant difference in the transpiration rate between the CT4 blueberry plants and the control group (Figure 3D).

F_v_/F_m_ reflects the maximum photochemical efficiency of PSII to use light energy and electron transfer. The root pruning treatment significantly reduced F_v_/F_m_ only 7 days after pruning. In the absence of root pruning, F_v_/F_m_ was 0.76 and dropped to 0.75 under CT4 treatment, which was 1.32% lower than the control. There was no significant difference in F_v_/F_m_ at 21 and 42 d after root pruning compared to the control (Figure 3E).

ΦPSII represents the actual photochemical quantum efficiency of PSII in plants under illumination, indicating the proportion of absorbed energy used for photochemical pathways relative to the total absorbed energy. ΦPSII values represent an important indicator of plant photosynthetic efficiency. After 7 days of treatment, 40% root pruning (CT4) ΦPSII values were significantly increased by 11.11% compared to the control. However, after 21 days of treatment, CT4 ΦPSII values were significantly lower, exhibiting a 19.05% reduction compared to the control, suggesting an inhibition of the photosynthetic rate as well as potential damage to the photosynthetic apparatus. At 42 days after treatment, there was no significant difference in ΦPSII, indicating potential recovery from the temporary damage to the photosynthetic apparatus (Figure 3F).

Non-equilibrium quenching (NPQ) reflects the plant’s ability to dissipate excess light energy into heat, reflecting the plant’s photoprotective capacity. The root pruning treatment significantly reduced NPQ only 7 days after pruning. The NPQ value of CT4 was reduced to 2.44, which was 18.94% lower than that of the control group, indicating that the plant converted less of the absorbed light energy into heat and allocated a greater proportion towards chemical energy conversion, thus increasing the efficiency of utilization for light energy. There was no significant difference in NPQ at 21 and 42 days after root pruning compared to the control (Figure 3G). Short-term (0–7 days) stomatal restriction induced by CT4 treatment reduced the CO_2_ supply, leading to a light energy redistribution contingency. The medium-term (7–21 days) recovery of stomatal function was accompanied by a sudden drop in ΦPSII, suggesting an impaired photosystem. The long-term (21–56 days) full recovery of photosynthetic parameters, combined with a large increase in leaf biomass, indicates a synergistic increase in photosynthetic efficiency and leaf expansion. Although root pruning is the main triggering factor, the observed response likely involves trade-offs in resource allocation.

### 3.4. Transcriptome Sequencing Analysis

**Figure 4:**
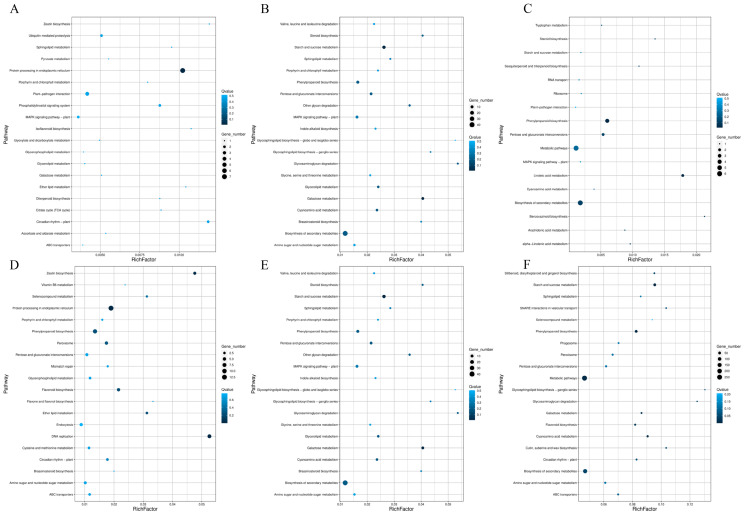
Enrichment of DEGs in KEGG pathways in blueberry leaves and roots during T1, T2, and T3. KEGG enrichment analysis of DEGs in blueberry leaves: CK-T1 vs. CT-T1 (**A**), CK-T2 vs. CT-T2 (**B**), and CK-T3 vs. CT-T3 (**C**). KEGG enrichment analysis of DEGs in blueberry roots: CK-T1 vs. CT-T1 (**D**), CK-T2 vs. CT-T2 (**E**), and CK-T3 vs. CT-T3 (**F**).

There were significant differences in the growth and photosynthesis of blueberry plants between CT4 treated and CK treated at 7 days (T1), 21 days (T2), and 42 days (T3) after root pruning. To identify differentially expressed genes (DEGs) associated with blueberry root pruning, CT4 was subsequently selected for subsequent transcriptome sequencing of leaf (L) and root (R) tissues at T1, T2, and T3 after root pruning (Figure 4). After the quality control filtering of raw sequencing data to obtain clean reads (Q30 ≥ 90%), these reads were compared to the *Vaccinium darrowii* reference genome using HISAT2, with a comparative efficiency of >91.26%. A total of 1774 DEGs were screened according to the screening criteria for DEGs (|log2FC| > 1 and *p* < 0.05). The distribution of DEGs across different comparisons was as follows: in leaf tissue, CK-L-T1 vs. CT-L-T1 yielded 53 DEGs (15 upregulated and 38 downregulated); CK-L-T2 vs. CT-L-T2 yielded 245 DEGs (184 upregulated and 61 downregulated); and CK-L-T3 vs. CT- L-T3 yielded 21 DEGs (13 upregulated and 8 downregulated). For the root system, CK-R-T1 vs. CT-R-T1 yielded 194 DEGs (141 upregulated and 53 downregulated); CK-R-T2 vs. CT-R-T2 yielded 106 DEGs (76 upregulated and 30 downregulated); and CK-R-T3 vs. CT-R-T3 yielded 1155 DEGs (886 upregulated and 269 downregulated).

KEGG pathway enrichment analysis was performed on DEGs generated at the three time points. Regarding KEGG pathway enrichment analysis, 53 DEGs were significantly overexpressed in 28 different KEGG pathways on day 7. The pathways with the highest levels of enrichment included metabolic pathways and the TCA cycle (Figure 4A). At day 21, 245 DEGs were identified as differentially expressed across 71 KEGG pathways, where the metabolic pathways with the highest enrichment levels were the secondary metabolite synthesis pathway and the carbon metabolism pathway (Figure 4B). By day 42, 21 DEGs exhibited significant enrichment in 17 KEGG pathways, including the secondary metabolite synthesis pathway and the starch and sucrose synthesis pathway (Figure 4C).

Regarding the enrichment analysis of KEGG pathways, on day 7, 194 DEGs were significantly overexpressed in 52 different KEGG pathways. The pathways with the highest enrichment levels included the metabolic pathway and the starch and sucrose metabolism pathways. This was closely followed by the carbon metabolism pathway (Figure 4D). On day 21, 106 DEGs were significantly enriched in 50 KEGG pathways, including the secondary metabolite synthesis pathway, starch and sucrose synthesis pathway, and carbon metabolism pathway (Figure 4E). On day 42, 1155 DEGs were significantly enriched in 118 KEGG pathways, including the synthesis pathway, starch and sucrose synthesis pathway, photosynthesis, and carbon metabolism pathway (Figure 4F).

### 3.5. Effect of Root Pruning on the Expression of STARCH, Sucrose, and Carbon Metabolism Pathway Genes in Blueberry Roots

**Figure 5:**
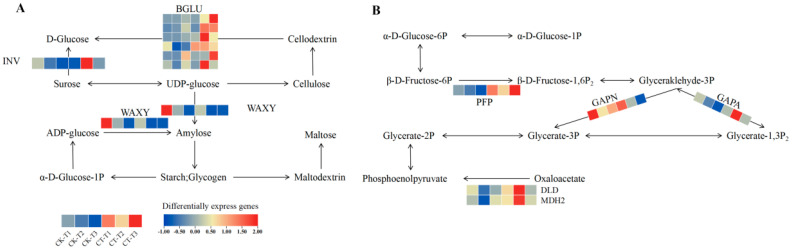
Analysis of metabolic pathways associated with vascular tissue development. Analysis of DEGs related to starch and sucrose metabolic pathways (**A**). Analysis of DEGs associated with carbon metabolic pathways (**B**). Used to synthesize UDP-glucose, a substrate for cellulose synthesis. The three stages of root development after blueberry root pruning are presented in the heatmap, from left to right, CK-T1 (CK-7d), CK-T2 (CK-21d), CK-T3 (CK-42d), CT-T1 (CT4-7d), CT-T2 (CT4-21d), and CT-T3 (CT4-42d). The data presented in the heatmap is the average of three biological replicates. Enzyme reactions associated with gene expression involving each step are presented on the corresponding arrows, containing glucoside hydrolase (BGLU), sucrose invertase (INV), and granule-bound starch synthase (WAXY). The gene expressions involved were BGLU (Vadar_g18328, Vadar_g18327, Vadar_g39897, Vadar_g39093, Vadar_g228 and Vadar_g25404), INV (Vadar_g16537), and WAXY (Vadar_g15976). (**B**) Analysis of DEGs related to carbon metabolic pathways. The data presented in the heatmap are the average of three biological replicates. Enzyme reactions associated with gene expression involving each step are presented on the corresponding arrows, containing (Diphosphate-dependent phosphofructokinase, PFP), (Glyceraldehyde-3-phosphate dehydrogenase, GAPN), (Glyceraldehyde 3-phosphate dehydrogenase, GAPA), (Dihydrolipoyl dehydrogenase, DLD), and (Malate dehydrogenase, MDH2). The gene expressions involved were PFP (Vadar_g33626), GAPN (Vadar_g28061), GAPA (Vadar_g39062), DLD (Vadar_g11578), and MDH2 (Vadar_g46714). CK, non-root pruning; CT, root pruning.

KEGG pathway analysis showed the significant enrichment of DEGs in the starch and sucrose metabolic pathway and the carbon metabolic pathway at three time points. These pathways provide substrates for cellulose biosynthesis by regulating UDP sugar synthesis. In the starch and sucrose metabolism pathways, the expression of genes encoding glycoside hydrolase (BGLU) and sucrose converting enzyme (INV) was consistently upregulated in the treatment group, with increased root development after root pruning compared to the control group, which significantly promoted UDP-sugar synthesis. In contrast, granule-bound starch synthase gene expression was repressed, leading to reduced starch accumulation and contributing to the preferential use of carbon sources for the preferential use in UDP sugar synthesis. The above changes in gene expression significantly elevated the rate of UDP-sugar synthesis, providing a sufficient substrate for cellulose synthesis (Figure 5A; Table S5).

Within the carbon metabolism pathway, all differentially expressed genes encoding diphosphate-dependent phosphofructokinase (PFP) were consistently upregulated at the T1, T2, and T3 phases in the treatment group compared with the control group. Conversely, all differentially expressed genes encoding glyceraldehyde-3-phosphate dehydrogenase (GAPN) and dehydrogenase (GAPN) were significantly downregulated. Genes encoding dihydrolipoyl dehydrogenase (DLD) and malate dehydrogenase (MDH2) were significantly upregulated in the T2 phase. All significant differential genes encoding glyceraldehyde 3-phosphate dehydrogenase (GAPA) were significantly upregulated at both T2 and T3. The changes in the expression of the above genes synergistically promoted the conversion of α-D-Glucose-6P to α-D-Glucose-1P, a direct precursor of UDP-glucose. The elevated synthesis rate of UDP-glucose may originate from the differential expression of these pathway genes, ultimately driving cellulose biosynthesis through an enhanced substrate supply (Figure 5B).

### 3.6. Effect of Root Pruning on the Expression of Transcription Factors for Vascular Tissue Development in Blueberry Roots

**Figure 6:**
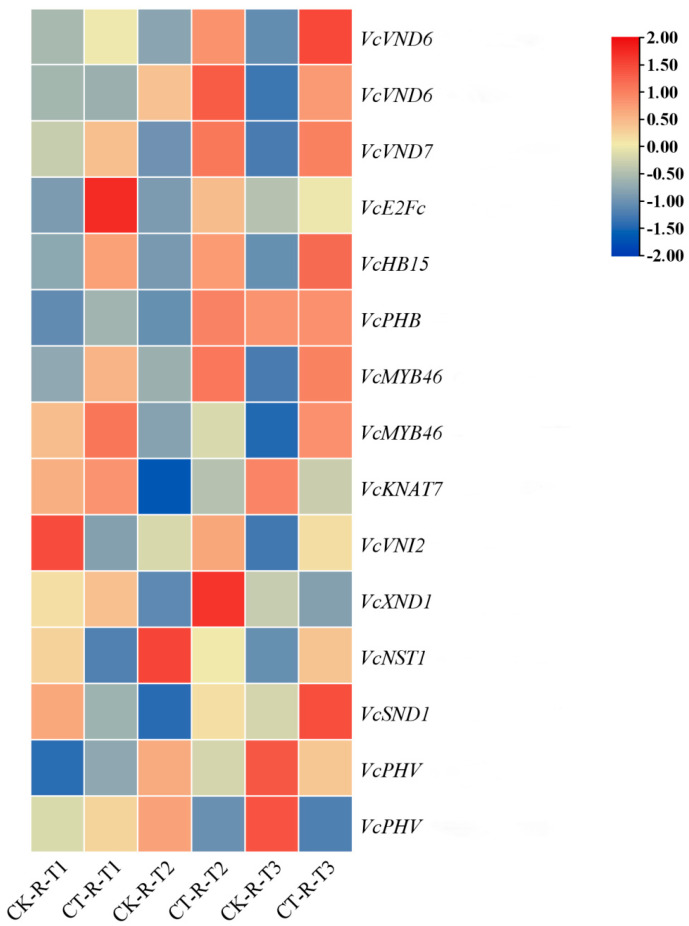
Heat map of transcription factors associated with root vascular tissue development after root pruning. The color scale bar in the top-right corner represents an increase (red), a decrease (blue), and no change (yellow) in gene expression. Sampling was performed 7 days (T1), 21 days (T2), and 42 days (T3) after root pruning, as indicated by the English subscripts in the figure. The gene expressions involved were *VcVND6* (Vadar_g5252), *VcVND6* (Vadar_g16528), *VcVND7* (Vadar_g26196), *VcE2Fc* (Vadar_g23325), *VcHB15* (Vadar_g37392), *VcPHB* (Vadar_g6161), *VcMYB46* (Vadar_g39307), *VcMYB46* (Vadar_g22345), *VcKNAT7* (Vadar_g5151), *VcVNI2* (Vadar_g45099), *VcXND1* (Vadar_g1180), *VcNST1* (Vadar_g9575), *VcSND1* (Vadar_g38770), *VcPHV* (Vadar_g3231), and *VcPHV* (Vadar_g41490). CK, non-root pruning; CT, root pruning.

Transcription factors such as *VcVND6*, *VcVND7*, and *VcE2Fc* are essential in vascular tissue development. Accordingly, we further characterized the expression of the homologous genes of these transcription factors in blueberry. Key TFs related to vascular tissue development were identified in *Arabidopsis* and *Populus trichocarpa* and anchored to related high-similarity transcription factors by homologous sequence comparison with blueberry amino acid sequences (Figure 6; Table S6). A phylogenetic tree was constructed by aligning the amino acid sequences of these TFs with the NAC, MYB, HD-ZIP, TALE, and E2F gene families of *Arabidopsis* and the NAC and MYB gene families of *Populus*, which showed that the 15 TFs exhibiting differential expression in blueberries shared a high degree of homology with the key TFs that regulate vascular tissue development in *Arabidopsis* and *Populus*. Most of the TFs involved in the biosynthetic pathways of lignin, cellulose, and xylan showed significant upregulation in the treated group compared with the control group. Among them, the expression of *VcVND6*, *VcVND7*, *VcE2Fc*, *VcHB15*, *VcPHB*, *VcMYB46*, and other TFs was significantly upregulated at T1, T2, and T3. *VcKNAT7*, as a direct target of *MYB46*, was significantly upregulated at both T1 and T2. Regarding the negatively regulated TFs, *VcVNI2* was significantly downregulated at T1, while *VcXND1* was significantly downregulated at T3. *VcVNI2* and *VcXND1*, as transcriptional repressors that negatively regulate vascular tissue development, were significantly downregulated at T1 and T3, respectively. *NST1* and *SND1*, the main regulators of secondary cell wall biosynthesis in fibers, were significantly upregulated by *VcNST1* at T3, while *VcSND1* was significantly upregulated at both T2 and T3. *VcPHV* was significantly upregulated at T1. In summary, positively regulated transcription factors were activated, and negatively regulated transcription factors were repressed after root pruning treatment. Root pruning drives the morphological remodeling and functional strengthening of vascular tissues in blueberry roots by reconfiguring the regulatory network of secondary cell wall synthesis.

### 3.7. Effect of Root Pruning on Gene Expression in Photosynthesis, Glycolysis, and Tricarboxylic Acid Cycle Pathways in Blueberry Leaves

**Figure 7:**
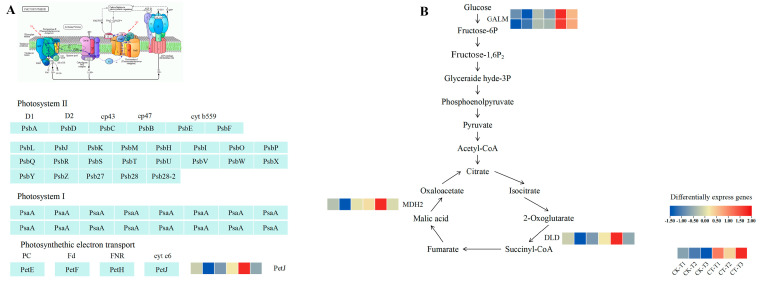
Analysis of metabolic pathways associated with root pruning and photosynthesis. (**A**) Analysis of DEGs associated with the photosynthetic pathway. The data presented in the heatmap is the average of three biological replicates. Enzyme reactions associated with gene expression involving each step are presented on the corresponding arrows containing cytochrome c6 and Pet J. The gene expression involved in this process was Pet J (Vadar_g7406). (**B**) Analysis of DEGs associated with carbon metabolism and tricarboxylic acid cycle pathways. The data presented in the heatmap are the average of three biological replicates. Enzyme reactions associated with gene expression involving each step are presented on the corresponding arrows, containing aldose 1-epimerase (GALM), dihydrolipoyl dehydrogenase (DLD), and malate dehydrogenase (MDH2). The genes involved were GALM (Vadar_g20155 and Vadar_g20154), DLD (Vadar_g11578), and MDH2 (Vadar_g46714). CK, non-root pruning; CT, root pruning.

KEGG pathway analysis revealed that a considerable number of DEGs were consistently upregulated across the three-time intervals in the photosynthesis pathway, the glycolysis and gluconeogenesis metabolic pathways, and the TCA cycle pathway. Glycolysis provides raw materials for the tricarboxylic acid cycle, while gluconeogenesis provides the material basis for glycolysis and the tricarboxylic acid cycle by synthesizing glucose through photosynthesis. Meanwhile, the energy released from the tricarboxylic acid cycle supports various life activities in plants, including growth and development. Consequently, this study conducted expression analysis on genes enriched in these three pathways, normalized gene expression levels to FPKM, and plotted expression heatmaps (Figure 7; Table S7). Within the above metabolic pathways, five differential genes encoding the five enzymes were monitored.

The expression profiles of DEGs enriched in the photosynthesis pathway at the three time points are shown in Figure 7A. One gene (encoding Vadar_g7406) showed significant upregulation in the treated group compared to the control group after both 7 and 21 days of treatment. One gene encoding cytochrome C6 (Pet J) exhibited upregulated expression at T2 and T3.

Figure 7B shows the expression profiles of DEGs enriched in glycolysis and gluconeogenesis and the TCA cycle at three-time points, with all genes upregulated in the treated group compared to the control group. All significantly differential genes encoding aldose 1-epimerase (GALM) were upregulated at T1, T2, and T3, while all significantly differential genes encoding malate dehydrogenase (MDH2) and dihydrolipoyl dehydrogenase (DLD) were upregulated at T1 and T2.

### 3.8. RT-qPCR Analysis

**Figure 8:**
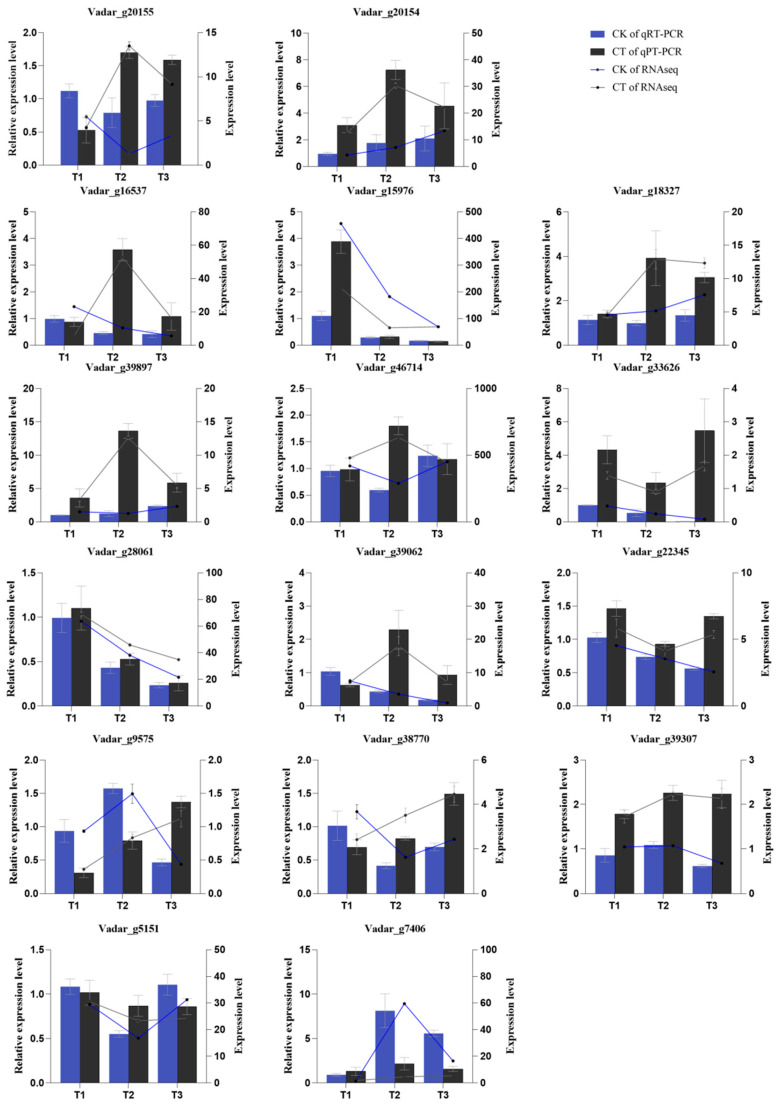
Validation of key genes related to vascular tissue development and photosynthesis in the roots and leaves of control and CT4 groups by qRT-PCR using actin as the reference gene. The column represents the results of qRT-PCR, with the coordinate axis on the left. The line represents the results of the transcriptome, with the coordinate axis on the right.

We next analyzed the expression levels of two genes involved in the photosynthesis pathway and 14 genes involved in the development of vascular tissues using fluorescence quantitative PCR (qRT-PCR) to validate the RNA-Seq results. The qRT-PCR results showed that the expression trends in the qRT-PCR closely aligned with those observed in the transcriptome sequencing data, confirming the consistency of gene expression trends despite variations in expression levels due to differences in experimental methods (Figure 8; Table S8).

## 4. Discussion

### 4.1. Proper Root Pruning Promotes Blueberry Plant Growth and Development

In this study, we found that moderate root pruning (CT4 treatment) significantly promoted the growth of aboveground parts of blueberry plants, as evidenced by the simultaneous increase in the leaf area, leaf dry weight, and total aboveground biomass (Figure 1B,D), whereas over-pruning (e.g., CT2/5/6/8 treatments) resulted in limited culm development and impeded the efficient accumulation of biomass, reflecting a typical “intensity-dependent” characteristic of the plant response to root damage [33]. From this perspective, moderate pruning (CT4) improves blueberry growth performance by triggering compensatory growth, while excessive pruning (CT2/5/6/8) leads to resource imbalance due to restorative consumption.

The significant increase in leaf biomass exhibited in the CT4 treatment group 56 days after root pruning may be closely associated with the compensatory strategy initiated by the plant through root-canopy signaling. Root damage may trigger the transport of stress signaling molecules through the xylem to the aboveground parts and activate photosynthesis-related metabolic pathways, thereby increasing carbon assimilation efficiency [34]. At the same time, the reduced respiratory consumption of older roots and the efficient nutrient uptake of newly developed fibrous roots after pruning might have disrupted the original “carbon-nitrogen balance”, leading to the preferential allocation of photosynthetic products to leaves, thus increasing the aboveground biomass. Unlike CT4, the other treatments (CT2/5/6/8) showed a significant decrease in basal diameter and no significant increase in total aboveground biomass, suggesting that they might be engaged in an “excessive damage-repair cycle”. The unique phenotype of the CT4 group (Figure 2F) may reflect the efficient water-conducting capacity of its developing fibrous root system. The lower lignification of the nascent root system may promote transpiration by increasing hydraulic conductivity, while the increase in leaf expansion pressure may drive the increase in leaf area by activating expansion-associated proteins to promote cell expansion [35]. This cascade of “optimization of root structure-enhanced water transport-enhanced leaf growth” provides a physiological basis for efficient resource utilization in the CT4 group.

Notably, the growth-promoting effect of CT4 was observed only 42 days after pruning (Figure 2E), aligning with the typical cycle of plant root remodeling [36]. During the early phase (7–21 days) (Figure 2B–E), the undifferentiated biomass mainly corresponded to the root regeneration process, whereas the exponential growth observed in the late phase (56 days) reflected a metabolic burst in the newborn root system following functional maturation. This temporal pattern suggests the importance of accounting for the physiological lag in plant response when evaluating the effects of root pruning, as short-term observations may underestimate the potential benefits.

### 4.2. Changes in the Expression of Genes Related to Vascular Tissue Development Led to Changes in Root Development

Plant roots and stems comprise vascular tissues. Our findings revealed that appropriate root-pruning treatments promoted root development. KEGG pathway analysis identified enzymes and key transcription factors related to cellulose and lignin synthesis. Cellulose, a major constituent of plant cell walls, provides structural integrity along with other cell wall polymers such as hemicellulose and lignin [37,38]. UDP glucose, the substrate for cellulose biosynthesis, can be produced by two pathways: one from the cleavage of sucrose catalyzed by SUS and the other from the phosphorylation of glucose-1-phosphate catalyzed by UGP [39,40,41]. In the cellulose synthesis pathway, all significantly differential genes encoding glycoside hydrolases were upregulated at T2 and T3; most of the differential genes involved in encoding sucrose-converting enzymes were significantly upregulated at T2. In plants, *VND6* and *VND7* genes are specifically expressed in vessels and play key roles in directing the differentiation of secondary xylem and protoxylem ducts, respectively [42]. In contrast to the function of *VNDs* in vessels, *NST1* and *NST3/SND1* are master regulators of secondary cell wall biosynthesis in fibers [43].

E2Fc, as a key upstream regulator of *VND6*, *VND7*, and other SCW biosynthesis genes, plays an important role in regulating the expression of these genes [44,45]. The present study revealed that following root pruning treatment, the expression of *VcVND6*, *VcVND7*, and *VcE2Fc* was significantly activated in conduit cells in the blueberry root system, exhibiting upregulation at T1, T2, and T3. Meanwhile, *VcNST1* was significantly up-regulated at T3, while *VcSND1* showed significant upregulation at both T2 and T3. These results suggest that proper root pruning can effectively promote the differentiation of secondary xylem and primary xylem conduits in the blueberry root system, which in turn exerts a profound effect on root development.

In addition, *VNI2*, a transcriptional repressor that interacts with *VND7*, can restrict the expression of vessel-specific genes regulated by *VND7* [46]. In contrast, the transcription factor *XND1* functions to negatively regulate xylem conduit differentiation [47]. Notably, *VcVNI2* was significantly downregulated at T1, whereas *VcXND1* was significantly downregulated at T3 after root pruning treatment, which further revealed the complex effects of root pruning on the regulatory network in plants.

On the other hand, HD Zip III genes are negatively regulated by highly conserved miRNAs, among which the three HD Zip III transcription factors (TFs), *PHB*, *PHV*, and *HB15*, are essential for xylem cell identification and secondary cell wall synthesis. The promoter of *PHB*, in particular, can be bound by *VND7*, thereby regulating its expression [48]. In addition, the overexpression of *MYB46* enhances lignin, cellulose, and xylan biosynthesis pathways and may act by regulating other SCW-associated TFs or by directly regulating SCW structural genes [49,50]. The results demonstrated that the expression of VcHB15, *VcPHB*, and *VcMYB46* was significantly upregulated in blueberry roots at T1, T2, and T3; *VcPHV* was significantly upregulated at T1. Notably, KNAT7, as a direct target of MYB46, is a class junction homology box (KNOX) protein and a common target of *MYB46* [51] and *SND1*. Prior research reported that the dominant inhibition of *KNAT7* significantly reduced SCW thickening in vessels and fibers, further demonstrating the important role of *MYB46* and its regulatory network in secondary cell wall biosynthesis [52]. In the present study, the expression of *VcKNAT7*, a direct target of *MYB46*, was significantly upregulated at both T1 and T2 in blueberry roots.

Based on the above findings, it can be inferred that changes in the expression of key transcription factors and enzymes related to vascular tissue development have significant effects on root growth and development. They affect root growth and development by regulating vascular tissue differentiation, thus helping plants to better adapt to different environmental conditions and nutritional requirements, but the specific pathways through which root pruning alter the expression of these genes need to be further explored.

### 4.3. Proper Root Pruning Promotes Plant Photosynthesis

Studies have shown that root pruning substantially impacts photosynthesis in leaves [53,54]. This study revealed the response of photosynthetic performance and related molecular mechanisms in blueberry plants to CT4 treatment over time. The net photosynthetic rate, stomatal conductance, intercellular CO_2_ concentration, and F_v_/F_m_ were significantly reduced at the beginning of the post-treatment period (T1), indicating a transient inhibition of photosynthesis. This short-term inhibition may have originated from impaired water uptake caused by root damage, which triggered stomatal closure to minimize water loss through transpiration. Meanwhile, the restricted CO_2_ supply directly limited carbon fixation in the Calvin cycle. In this regard, the slight decrease in F_v_/F_m_ indicated mild photoinhibition, whereas the significant decrease in NPQ indicated that the absorbed light energy was reallocated to photochemical reactions instead of heat dissipation, which may represent an adaptive mechanism to optimize light use under stress conditions.

At T2, photosynthetic parameters gradually recovered to the control level; however, the significant decrease in ΦPSII indicated that the actual photochemical efficiency of PSII was suppressed. This phenomenon may be related to the imbalance between light energy uptake and carbon metabolism after the recovery of carbon assimilation capacity at the mid-term stage. Potential photosystem damage may have led to a decrease in the efficiency of the photochemical electron transport chain. Nonetheless, gene expression analysis revealed that glycolysis and tricarboxylic acid cycle (TCA)-related genes (e.g., MDH2, DLD) were consistently upregulated, suggesting that the plants provided energy support for photosystem repair through enhanced respiratory metabolism.

At T3, the complete restoration of photosynthetic function coincided with a sustained upregulation of photosynthesis-related genes (e.g., encoding cytochrome C6), potentially enhancing the efficiency of electron transfer between PSII and PSI, thereby facilitating carbon assimilation. Meanwhile, the overexpression of glycolysis (*GALM*) and TCA cycle (*MDH2*, *DLD*) genes emphasizes a metabolic redistribution strategy that enhances carbon substrate supply. Specifically, gluconeogenesis may compensate for carbon deficiency during early stress by replenishing photosynthetic intermediates, whereas the activation of the TCA cycle provides energy and biosynthetic precursors for leaf expansion and organ formation. This dynamic coordination between photosynthetic carbon assimilation and respiratory consumption ensures efficient energy rebalancing, which ultimately supports biomass accumulation.

In summary, our findings suggest that moderate root pruning induces a staged adaptive response: initial stomatal closure and photochemical adjustments are followed by metabolic upregulation to mitigate photoinhibition, which ultimately synergizes to improve photosynthetic efficiency and plant growth.

### 4.4. Coordinated Development of Aboveground and Belowground Plant Structures

**Figure 9:**
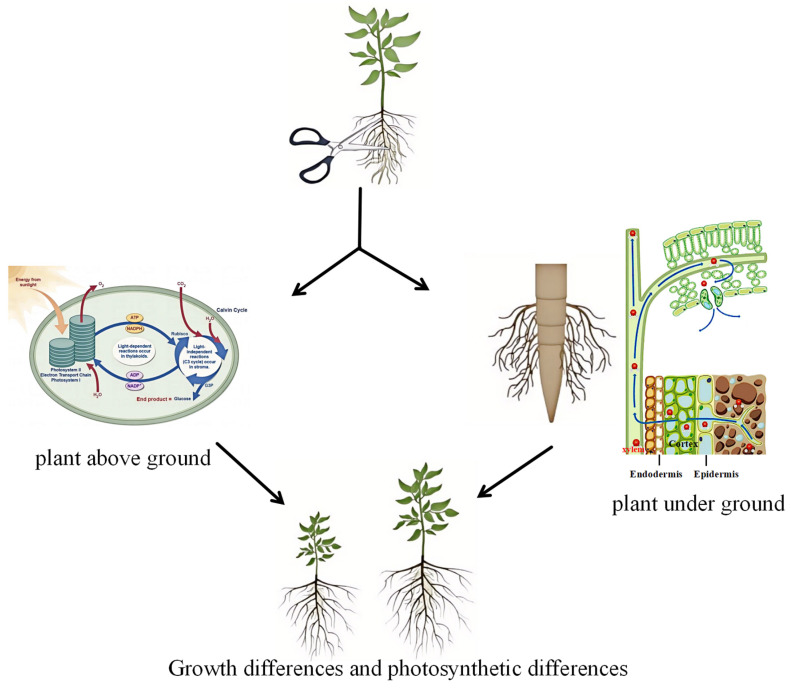
Schematic illustration of the coordinated development of the aboveground and underground parts of blueberry after root pruning.

Over the years, numerous studies have shown that proper root pruning can stimulate plant growth as plants may enhance nutrient uptake and utilization efficiency to promote physiological repair and sustained plant growth, thereby mitigating, to some extent, the detrimental effects of injury [55,56]. In this study, increased carbon allocation was required for belowground development, and CT4 treatment significantly upregulated photosynthesis-related genes, which led to enhanced photosynthesis in the aboveground leaves of blueberry plants. Accordingly, a greater proportion of photosynthesis products was translocated to the roots through the vascular tissues, causing root development (Figure 9). Root development in blueberry plants requires leaf photosynthetic activity for the accumulation of carbon resources.

Although this study provides mechanistic insights into root pruning responses, several limitations should be considered. First, our experiments used a single blueberry variety (*Vaccinium corymbosum* “Eureka”), which may limit the generalizability of the results to other varieties with different genetic backgrounds. Second, while the controlled environment of the greenhouse is crucial for standardizing variables such as light, temperature, and humidity, it differs from field conditions. Soil heterogeneity, biotic stress, and climatic fluctuations in field environments may modulate pruning outcomes. Future studies should validate these findings across different varieties, field environments, and integrative omics approaches to optimize pruning protocols for commercial applications.

## 5. Conclusions

This study demonstrates that moderate root pruning (removing 40% of root mass) triggers a coordinated molecular–physiological reprogramming that optimizes blueberry growth. Key findings include the following: vascular remodeling drives root regeneration; at 7 days, 21 days, and 42 days, the sustained upregulation of vascular transcription factors (*VcVND6/7*, *VcSND1*, *VcMYB46*) promoted xylem differentiation (Figure 6) and enhanced the transport of photosynthetic products to pruned roots. The downregulation of inhibitory factors (*VcVNI2*, *VcXND1*) releases developmental constraints, accelerating vascular reconnection. Metabolic reprogramming drives compensatory growth: UDP-glucose accumulation mediated by BGLU/INV activates the cellulose synthesis pathway (Figure 5A), while starch metabolism shifts toward structural carbohydrate production (WAXY downregulation). Carbon flux redistribution significantly increases the leaf biomass by 42 days (Figure 1D), providing resources for root reconstruction. Photosynthetic resilience supports source-sink coordination: temporary photosynthetic inhibition at 7 days (Figure 3A, E) is restored by enhanced electron transport mediated by PetJ (Figure 7A) and respiratory compensation (upregulation of GALM/MDH2) by 21 days. Long-term leaf expansion synergizes with vascular efficiency to enhance the carbon output capacity.

## Data Availability

The data presented in this study are available on request from the corresponding author.

## Supplementary Materials

The following supporting information can be downloaded at: https://www.mdpi.com/article/10.3390/plants14152269/s1, Figure S1: The complete root system scanning image and partial cropped images of the CK group at 7 days after root pruning; Figure S2: Actual image of root culture device; Figure S3: Illustration of root pruning methods. Table S1: Primer sequences for quantitative PCR; Table S2: Raw data of the bar chart in Figure 1; Table S3: Raw data of the bar chart in Figure 2; Table S4: Raw data of the bar chart in Figure 3; Table S5: Raw data of the bar chart in Figure 5; Table S6: Raw data of the bar chart in Figure 6; Table S7: Raw data of the bar chart in Figure 7; Table S8: Raw data of the bar chart in Figure 8.
