# Supplementary material for: The Molecular Mechanism and Effects of Root Pruning Treatment on Blueberry Tree Growth
Source: Plants (Basel). 2025 Jul 23;14(15):2269. doi: 10.3390/plants14152269 (PMC12348567; doi:10.3390/plants14152269)
Supplement: Supplementary file 1 [file plants-14-02269-s001.zip › Supplementary information 2.pdf]

Supplementary information

Figure S1. The complete root system scanning image and partial cropped images of the CK group at 7 days after root pruning.

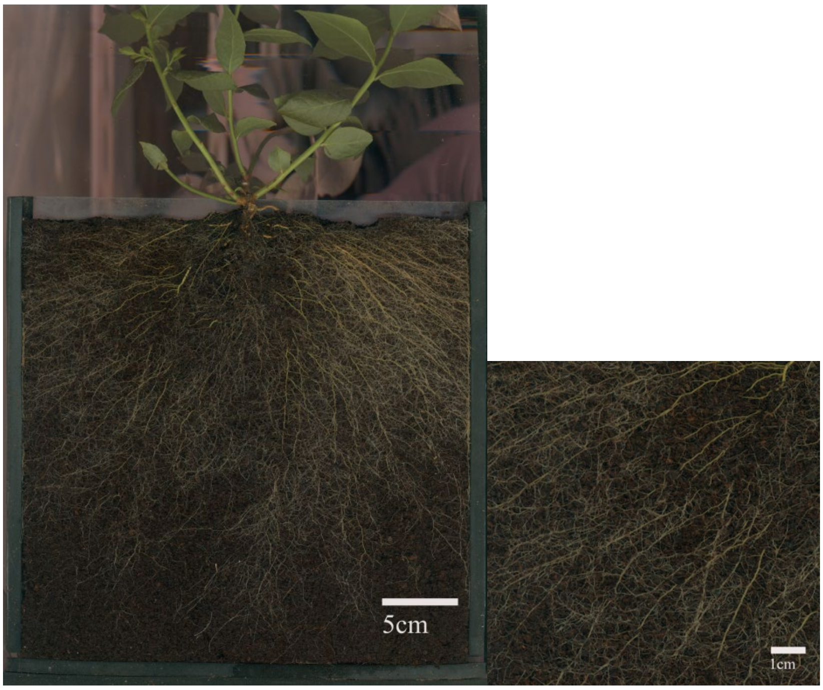

The complete root system scanning image and partial cropped images of the CK group at 21 days after root pruning.

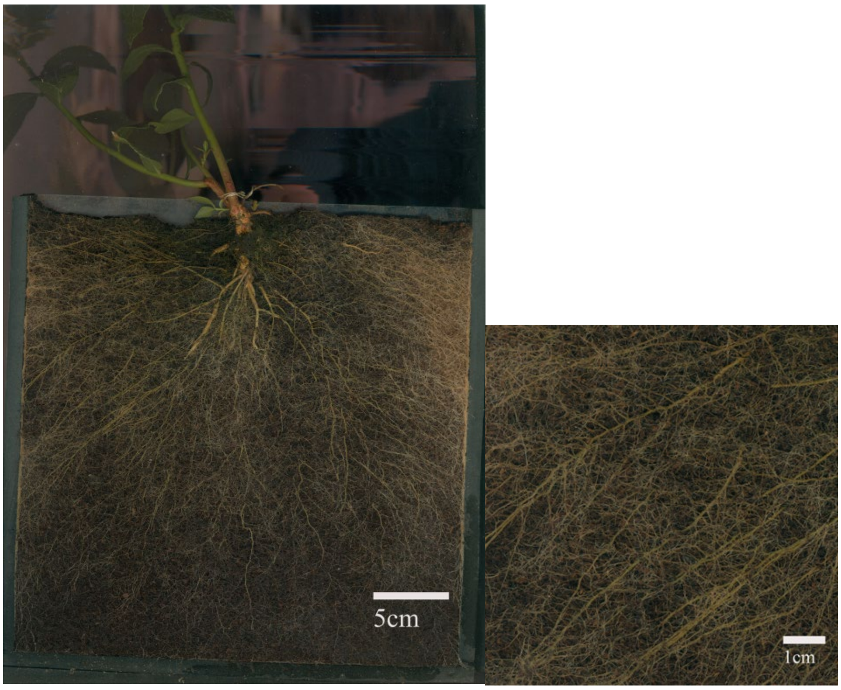

The complete root system scanning image and partial cropped images of the CK group at 42 days after root pruning.

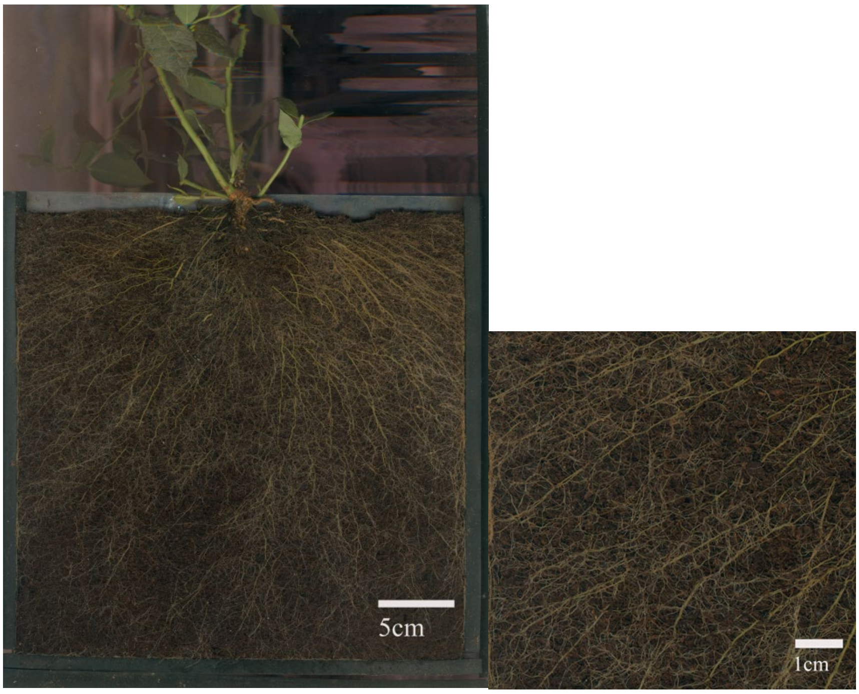

The complete root system scanning image and partial cropped images of the CT4 group at 7 days after root pruning.

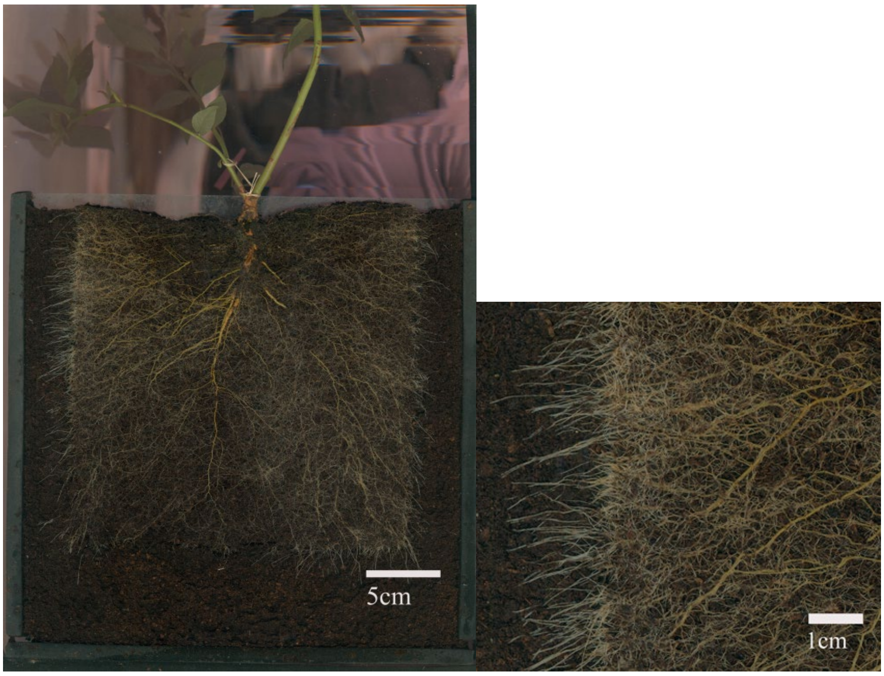

The complete root system scanning image and partial cropped images of the CT4 group at 21 days after root pruning.

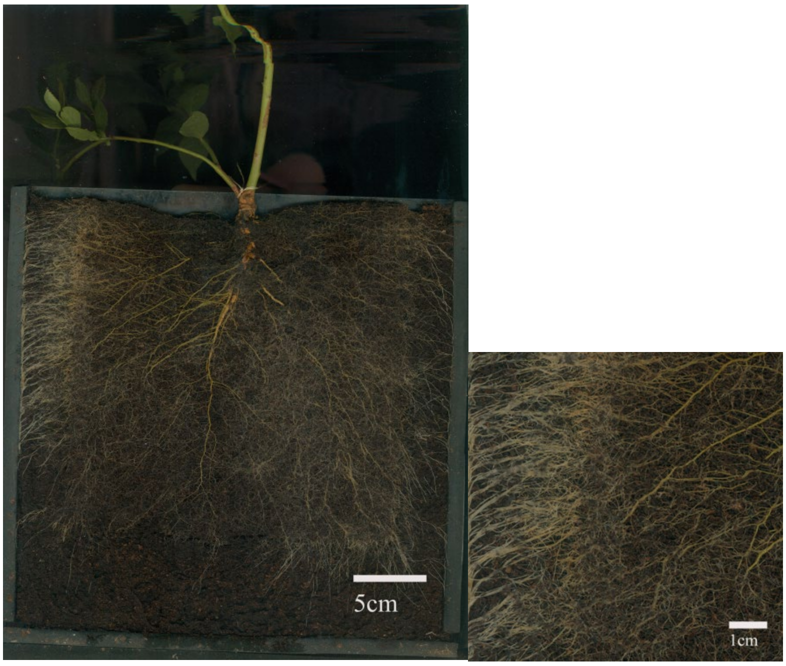

The complete root system scanning image and partial cropped images of the CT4 group at 42 days after root pruning.

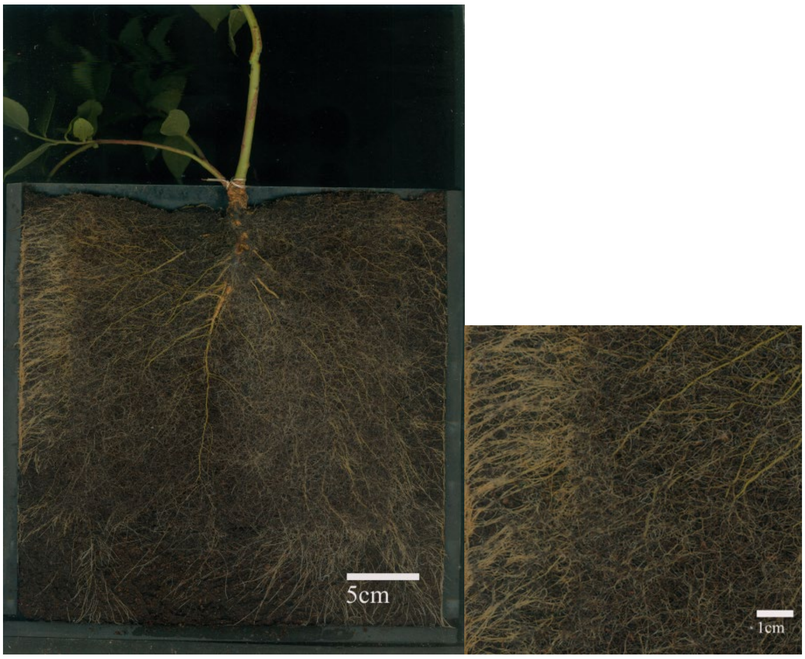

Figure S2. Actual image of root culture device

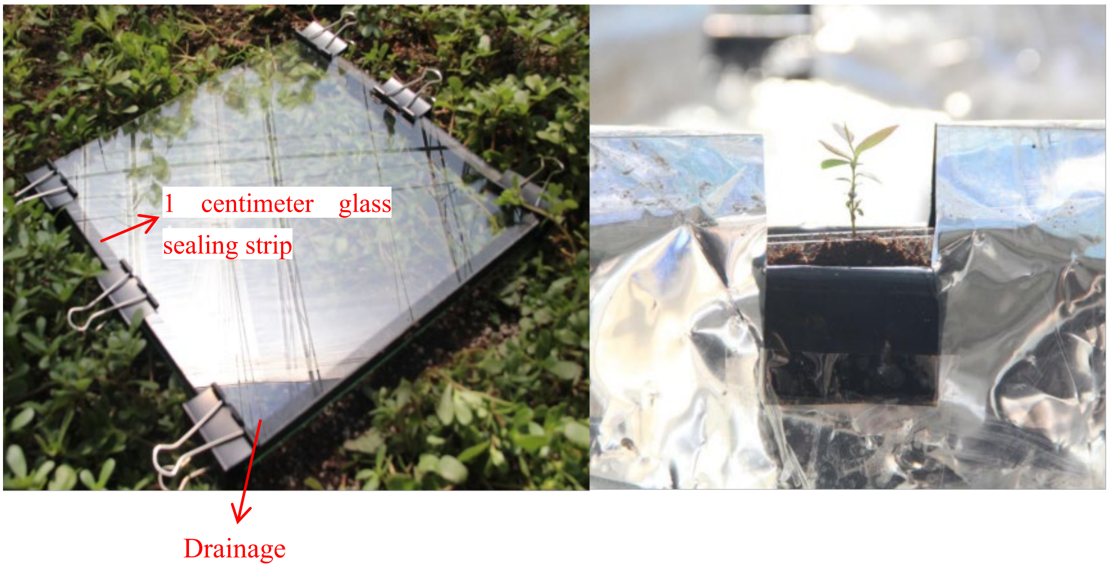

Figure S3. Illustration of root pruning methods

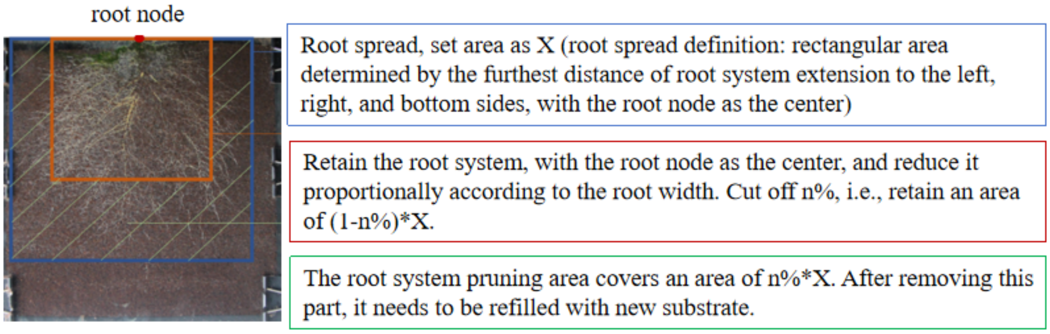

Note: Left image: Conceptual diagram of root pruning; Right image: Practical diagram of root pruning, showing pruning of 40% of the root system.
